# Psychometric Properties of the QoL-ME: A Visual and Personalized Quality of Life Assessment App for People With Severe Mental Health Problems

**DOI:** 10.3389/fpsyt.2021.789704

**Published:** 2022-01-05

**Authors:** David C. Buitenweg, Dike van de Mheen, Hans A. M. van Oers, Chijs van Nieuwenhuizen

**Affiliations:** ^1^GGzE Institute for Mental Health Care, Eindhoven, Netherlands; ^2^Tranzo Scientific Center for Care and Wellbeing, Tilburg School of Social and Behavioral Sciences, Tilburg University, Tilburg, Netherlands

**Keywords:** quality of life, psychometric evaluation, validity, reliability, responsiveness, visual assessment, e-mental health

## Abstract

**Background:** Quality of Life (QoL) assessment in people with severe mental health problems may benefit from improved personalization and accessibility. Therefore, an innovative, digital, visual, and personalized QoL assessment app for people with severe mental health problems was recently developed: the QoL-ME. The aim of this study was to evaluate the psychometric quality of the QoL-ME by assessing its reliability, validity, and responsiveness.

**Methods:** To examine the reliability of the QoL-ME, the internal consistency of its subscales was assessed using Cronbach's Alpha. Correlations between the QoL-ME and the MANSA were computed to appraise the construct validity of the QoL-ME. Internal responsiveness was evaluated using the standardized response mean and external responsiveness was investigated using hierarchical regression.

**Results:** Cronbach's Alpha's of the subscales of the QoL-ME ranged between 0.5 and 0.84. In accordance with expectations, the language-based core version of the QoL-ME correlated strongly (*r* = between 0.55 and 0.76) with the MANSA, whilst the picture-based additional modules of the QoL-ME correlated moderately (*r* = 0.3) with the MANSA. The standardized response mean was 0.23 and the regression model revealed a coefficient β of −0.01.

**Conclusions:** The QoL-ME has adequate psychometric properties. In comparison with similar pictorial instruments, both the QoL-ME's reliability and validity can be considered as sufficient. The results indicate that the responsiveness of the QoL-ME is insufficient. Additional research is needed to evaluate and potentially modify the instrument to improve its responsiveness.

## Introduction

Quality of life (QoL) is an essential patient-reported outcome in mental health services (1–3). Subsequently, a number of instruments to assess the QoL of people with severe mental health problems have been developed (3, 4). These instruments, such as the Lancashire Quality of Life Profile [LQoLP; (5)] and the Manchester Short Assessment of Quality of Life [MANSA; (6)], communicate using language and generally assess QoL on the basis of a fixed set of life domains, such as “Social relations,” “Living situation,” and “Finances” (5, 7). Respondents are required to respond to a statement or question by selecting one of multiple Likert options. This conventional approach to QoL assessment faces three important challenges. First, recent research reemphasizes the subjective nature of QoL, as the concept is shaped by individual values and priorities (8–10). Respecting this subjective aspect of QoL requires a more personalized assessment. Second, existing QoL instruments depend on verbal, language-based communication. Research indicates that this language-based approach may not be optimal for every individual with severe mental health problems (11, 12). Visual communication may provide a suitable alternative to language-based methods as it requires less processing and is more intuitive than verbal communication (13–15). Third, given the continuing digitalization of society and mental healthcare (16, 17) it is vital to explore the potential of digital applications in QoL assessment. Examples of characteristics of digital applications that may benefit QoL assessment include their flexibility (18, 19) and multimedia compatibility (20).

In response to these challenges in QoL measurement, a new digital QoL assessment app has recently been developed: the QoL-ME (21). The QoL-ME is a digital QoL assessment app that utilizes a personalized and visual assessment approach. The app consists of two main components: a core version and additional modules. The core version involves a few mandatory QoL domains that every respondent has to answer. In addition, respondents are free to select any combination of eight additional modules and only answer questions on their modules of choice. This structure, involving both a mandatory core version and optional additional modules, makes the QoL-ME a flexible QoL assessment app (21).

The QoL-ME was developed co-creatively together with patients (20). A usability evaluation, that was part of the development, revealed good to excellent usability scores (21). Participating patients were enthusiastic regarding the visual assessment approach employed in the QoL-ME and welcomed the opportunity to select QoL domains based on their personal preferences (21). No conclusion regarding the utility of the QoL-ME can be drawn, however, without an evaluation of its psychometric quality. A psychometric evaluation of the QoL-ME is of special importance in light of the visual assessment approach employed in the QoL-ME. This approach does not depend on respondents' language proficiency and is more intuitive (22). At the same time, visual information also tends to be more ambiguous than verbal information (23, 24). This ambiguity may have consequences for the validity and reliability of the QoL-ME. In addition, insight into the responsiveness of the QoL-ME is needed. A responsive QoL instrument reflects true changes or differences in QoL (25, 26). Sufficient reliability, validity, and responsiveness are essential qualities if the QoL-ME is to be of use in the context of scientific research and clinical practice.

Therefore, the aim of this study is to investigate the psychometric quality of the QoL-ME. To this end, the reliability, construct validity and responsiveness of the QoL-ME are investigated.

## Methods

### Participants

In this study, samples from three populations of people with severe mental health problems were included: (1) people with severe psychiatric problems, (2) people treated in forensic psychiatry, and (3) people who are homeless. These groups may have difficulty with traditional language-based QoL assessment due to experiencing fewer educational opportunities (27–29), co-occurring intellectual disabilities (29–31), and compromising psychiatric symptoms (11, 12).

Six societal organizations collaborated in a consortium to facilitate this study, including a multimodal day treatment center for multi-problem young adults, a hospital for forensic psychiatry, a mental health institution, a day center for people who are homeless and two research institutions focusing on lifestyle, homelessness and addiction. A group of 121 participants was recruited with the help of the consortium.

### Design

To assess the reliability, validity, and responsiveness of the QoL-ME, a quantitative longitudinal design was used. Participants were asked to fill out the QoL-ME every month during 6 months, leading to a maximum of seven measurements. The intermediate assessments served to investigate respondents' QoL-trajectories, which lies outside the scope of this article. Therefore, only the results gathered at the first measurement and final measurement will be discussed. During the first measurement (t0), participants also filled out the MANSA (11) and were asked a number of demographic questions. During the final measurement (tfinal), participants filled out the MANSA again. For practical reasons, roughly a third of participants (*n* = 39), who were included later in the study, had their final assessment after 4 months instead of 6 months. A one-way ANOVA was used to assess whether having a final assessment after 4 or 6 months had a significant effect on scores on both the QoL-ME and the MANSA at the first (2 ANOVA's) and final (2 ANOVA's) measurement. None of the four analyses returned a significant result. All final measurements were therefore taken together.

### Measures

#### The QoL-ME

Previous studies revealed a difference in universal QoL domains between (forensic) psychiatric patients on the one hand and people who are homeless on the other hand. Therefore, the QoL-ME contains two core versions (21, 32, 33). The content of both core versions and the additional modules is described in the following paragraphs.

The first core version targets people with (forensic) psychiatric problems and includes three domains of the LQoLP (5, 7): “Living situation,” “Safety,” and “Finances.” A recent study indicates the universality of these three domains (34), based on their high univariate entropies. Both “Living situation” and “Finances” are assessed using four items, whilst the domain “Safety” comprises five items. The first core version therefore contains 13 items. Examples of items included “How satisfied are you with the amount of money you make?” and “How satisfied are you with your general personal safety?”. The 7-point Likert scale used to assess these items ranges from “cannot be worse” (1) to “cannot be better” (5) and is identical to the scale used in the LQoLP (5, 7).

The second core version targets homeless people and covers two domains regarding meaning in life, which is especially important for homeless people (32, 33). The second core version consists of the Dutch version of the Meaning in Life Questionnaire [MLQ; (35)], a 10-item measure that assesses both the presence of meaning in one's life and the search for meaning in life (35). Examples of MLQ items include “My life has a clear sense of purpose,” and “I am searching for meaning in my life.” The MLQ also uses a seven-point Likert scale, ranging from “completely disagree” (1) to “completely agree” (5).

The additional modules for both core versions serve to ensure the personalization of the QoL-ME. The QoL-ME contains eight additional modules, all of which correspond to a domain of QoL: (1) Support and Attention, (2) Social Contacts, (3) Happiness and Love, (4) Relaxation and Harmony, (5) Leisure, (6) Lifestyle, (7) Finances, and (8) Health and Living. Users were free to select any combination of these eight modules by indicating whether the module was important or unimportant for them (see Supplementary Material 2). The eight QoL domains were identified in a visual concept mapping study of the QoL of people with severe mental health problems (36). Domains are assessed using two to four visual items. Every visual item contains three pictures that together depict an aspect of QoL. Users respond to these items using a Visual Analog Scale (VAS scale) with visual anchors. VAS scores range between 0 and 100. The VAS button was placed in the middle of the scale and had to be moved before the respondent may proceed to the next item.

The core version of the QoL-ME is especially useful in contexts where group-level data are of interest, such as comparisons of the QoL of different client populations. The additional modules are especially suitable for use in individual care planning. An introductory video of the QoL-ME and a video impression can be found in Supplementary Materials 1, 2. In addition, the development and content of the QoL-ME are described in detail elsewhere (21).

#### The MANSA

The MANSA is a shorter and slightly altered version of the LQoLP. The MANSA was developed by Priebe and colleagues (6). Van Nieuwenhuizen and colleagues (5, 37) developed an authorized Dutch version of the MANSA. The Dutch MANSA consists of 16 items, of which 12 assess the subjective QoL of respondents. The remaining four items measure objective circumstances. The objective items cover a respondents' circumstances (“In the past week have you visited with a friend?”), whilst the subjective items involve respondents' satisfaction with several life domains (“How satisfied are you with how well-off you are financially?”). The psychometric properties of the (Dutch version of the) MANSA were investigated extensively in multiple studies (37). In these studies, the reliability of the MANSA (Cronbach's alpha) ranged between 0.75 and 0.84. Convergent validity between the LQoLP and the MANSA ranged between 0.65 and 0.97 (37).

#### Demographic and Background Variables

In addition to the QoL-ME and the MANSA, participants were asked to fill out a number of basic demographic questions regarding their gender, age, cultural background, and employment status.

### Procedure

In the first assessment, participants contributed either in person, or on-line, depending on whether participants required personal assistance. Contacts at the consortium institutions approached potential participants using flyers and an information letter. Once participants indicated their interest in contributing to this study, they received an e-mail containing additional information on the study, and a detailed outline of what was expected of them. Moreover, the e-mail contained links to the QoL-ME and to Qualtrics; an online survey program used to administer the MANSA and the demographic questions. Once participants had filled out the online questionnaires, they received a €10 gift voucher by post. Alternatively, an appointment between researcher and participant was scheduled. During that appointment, the researcher provided additional information regarding the study, and outlined what was expected of the participant. Next, participants filled out the demographic items, the MANSA and the QoL-ME. Once all the questionnaires were filled out, participants received a €10 gift voucher. The procedure for the final measurement was similar to the procedure of the first assessment, but involved only the QoL-ME and the MANSA. Upon completing the last assessment, all participants received an additional €20 gift voucher.

### Statistical Analysis

Total scores on the MANSA were computed using the method described by Van Nieuwenhuizen et al. (37). To calculate a total score for the QoL-ME, mean scores were computed for every domain included in the core version and additional modules selected by respondents. As the core version involves a 7-point Likert scale and the additional modules uses a 0–100 VAS scale, all scores on the additional modules were transformed using the following formula: new score = (VAS score/100) ^*^ 7). Subsequently, the mean of all the domain scores was calculated to arrive at a total score.

To assess the reliability of the QoL-ME, the internal consistency of the subscales of the QoL-ME was evaluated using Cronbach's Alpha. Based on the size of Cronbach's Alpha, internal consistency was considered “excellent” (α ≥ 0.9), “good” (0.9 > α ≥ 0.8), “acceptable” (0.8 > α ≥ 0.7), “questionable” (0.7 > α ≥ 0.6), “poor” (0.6 > α ≥ 0.5) or “unacceptable” (0.5 > α) (38). The construct validity of the QoL-ME was evaluated based on the size of the correlation between scores on the QoL-ME and the MANSA at t0. The correlation between the core version for people with psychiatric problems and the MANSA was expected to be strong [>0.5; (39)] as both measures employ a language-based assessment approach. Note: As the core version for homeless people was not based on the LQoLP but on the MLQ. Therefore, we could not test the internal validity of this core version. The correlation between the QoL-ME's additional modules and the MANSA was expected to be medium sized [>0.3 and <0.5; (39)]. To further examine the validity of the visual assessment approach employed in the additional modules, correlations were computed for pairs of items of the additional modules and their corresponding MANSA items. Lacking fully objective criteria, this was done for items of the additional modules that have a parallel item in the MANSA. Pearson correlations were computed for six pairs of items, which are provided in Table 5. These correlations were also expected to be of medium size (>0.3 and <0.5).

In this study, both the internal responsiveness and the external responsiveness of the QoL-ME were of interest. Internal responsiveness pertains to the ability of a measure to change over a given time frame (40). The internal responsiveness of the QoL-ME was assessed using the Standardized Response Mean (SRM). The SRM is a type of effect size and is computed by dividing the mean change score (tfinal - t0) with the standard deviation of this mean change score (40). We expected a moderate SRM of around 0.5. External responsiveness is assessed by relating change on the measure of interest to change on an established measure (40). A hierarchical regression model was computed to assess the external responsiveness of the QoL-ME. In this model, QoL-ME scores at t0 served as the independent variable, whilst the MANSA score at tfinal was the dependent variable. MANSA score at t0 was used as a control variable. The regression coefficient β was used to assess the external responsiveness.

## Results

### Participants

A total of 121 participants agreed to contribute to this study and filled out the demographic items, the QoL-ME and the MANSA at the first measurement (t0). Seventy-two participants (59.5%) filled out the core version for (forensic) psychiatric patients. The group that filled out the core version for people who are homeless included 49 participants (40.5%). Participants' ages ranged between 17 and 66 with an average of 39.6 (SD = 14.9). A little over 70 percent of participants was male and 42.1 percent had a Dutch cultural background. Additional demographic characteristics are provided in Table 1. Eighty-one participants contributed to both t0 and tfinal (responders), whilst forty participants completed t0 but not tfinal (non-responders). Statistical analyses revealed that responders (*n* = 81) were significantly older (10.6 years) than non-responders (*n* = 40): *t*_(117)_ = 3.72, *p* < 0.01. A Chi-square test revealed no significant differences in the distribution between the two core versions for responders and non-responders: χ^2^ (1, *N* = 121) = 1.83, *p* = 0.176. Moreover, the groups did not differ significantly on other demographic characteristics including sex, cultural background, educational level or occupational status.

**Table 1:** Participants' demographic characteristics (*N* = 121).

Average age (SD)	39.6 (14.9)
Range age	17–66
Males (%)	85 (70.2)
**Cultural background**	
Dutch (%)	51 (42.1)
Western (%)	5 (4.1)
Non-western (%)	65 (53.7)
**Level of education^x^**	
Basic (%)	42 (34.2)
Intermediate (%)	65 (54.1)
Higher (%)	6 (5.0)
Unknown (%)	8 (6.7)
**Occupational status**	
Paid employment/Volunteer work (%)	50 (41.3)
Unemployed (%)	71 (58.7)

x *categorized based on the standard education classification (SOI−2016) of the Dutch CBS*.

### QoL-ME

Mean scores on the domains of both core versions of the QoL-ME can be found in Table 2.

**Table 2:** Mean scores on the domains of the core versions of the QoL-ME at t0 (*N* = 121), including standard deviations and range.

	**Mean score (SD)**	**Range**
**Core version homeless people (*****n*** **=** **49)**		
Searching for meaning	4.67 (1.18)	1.2–7
Presence of meaning	5.39 (0.99)	3.4–5.8
**Core version (forensic) psychiatric patients (n** **=** **72)**		
Living situation	5.02 (1.09)	1.75–7
Safety	5.15 (0.93)	2.8–7
Finances	3.95 (1.06)	1–7

Table 3 indicates how frequently the eight additional modules were selected at t0. This frequency ranged between 97 (80.2%) for the modules Social relations and Lifestyle and 111 (91.7%) for the modules Relaxation and harmony and Health and living. On average, respondents completed 6.9 additional modules (range = 2–8). Mean scores at t0 on the items of the additional modules of the QoL-ME are provided in Supplementary Material 3. Mean module scores are displayed in Table 3.

**Table 3:** Overview of the number of selections, mean scores at t0 and internal consistency of the eight additional modules (*N* = 121).

**Additional module**	**# selections**	**% of participants**	**Mean score (SD)**	**Internal consistency**
Support and attention	110	90.9	74.1 (21.9)	α = 0.69
Social relations	97	80.2	73.16 (22.3)	α = 0.78
Happiness and love	105	86.8	74.1 (22.2)	α = 0.84
Relaxation and harmony	111	91.7	77.34 (18.2)	α = 0.76
Leisure	103	85.1	72 (19.8)	α = 0.5
Lifestyle	97	80.2	76 (20.9)	α = 0.69
Finances	105	86.8	62.72 (26.7)	α = 0.70
Health and living	111	91.7	70 (23.5)	α = 0.63

### MANSA

The average total score on the MANSA at t0 was 4.52 (SD = 0.86). Analysis revealed good internal consistency for the MANSA in this sample: Cronbach's Alpha = 0.84.

### Reliability

The internal consistency of the three domains of the core version for people with (forensic) psychiatric problems was α = 0.74. (Finances), 0.76 (Living situation) and 0.83 (Safety). The internal consistency of the MLQ in this sample was α = 0.74. The internal consistency of the eight additional modules of the QoL-ME, computed using Cronbach's alpha, is provided in Table 3. Alpha's ranged between 0.50 (domain Leisure) and 0.84 (Domain Happiness and love).

### Validity

Correlations between the three domains of the QoL-ME core version for people with (forensic) psychiatric problems and their corresponding MANSA-domains were *r* = 0.55 (Living situation), *r* = 0.62 (Safety) and *r* = 0.76 (Finances). All correlations were significant (*p* < 0.01). Mean total scores for the MANSA and the additional modules of the QoL-ME correlated at *r* = 0.30, *p* < 0.01. The correlations between the six pairs of QoL-ME and MANSA items are provided in Table 4. Correlations ranged between 0.15 (Finances) and 0.39 (Living situation).

**Table 4:** Pearson correlations between six pairs of items of the additional modules of the QoL-ME and corresponding MANSA items.

**Item QoL-ME^a^**	**Item MANSA**	**Correlation (*n*)**
Support and attention item 1	Friendships	0.29^*^ (96)
Social relations item 3	Family relations	0.23^*^ (93)
Leisure item 1	Leisure	0.19 (100)
Finances item 1	Finances	0.15 (101)
Health and living item 1	Living situation	0.39^*^ (64)
Health and living item 2	Physical health	0.36^*^ (109)

a *The content of the items of the additional modules of the QoL-ME is provided in Supplementary Material 3*.

* *Significant correlation at p < 0.05*.

### Responsiveness

An overview of the mean scores on MANSA and QoL-ME at t0 and tfinal is displayed in Table 5. The SRM of the QoL-ME was 0.23. The hierarchical regression model revealed a regression coefficient of B = −0.01, *t*_(77)_ = −0.21, *p* = 0.83.

**Table 5:** Mean total scores for the QoL-ME and MANSA at t0 and tfinal.

	**t0 (*n* = 81)**	**tfinal (*n* = 81)**	**Δ scores**
QoL-ME (SD)	4.85 (0.91)	4.73 (0.94)	−0.12 (0.48)
MANSA (SD)	3.78 (0.66)	3.96 (0.6)	0.17 (0.37)

## Discussion

### General Discussion

In this study, the psychometric properties of the QoL-ME were assessed. The results show satisfactory reliability for most of the subscales of the core version and additional modules of the QoL-ME. In addition, the QoL-ME has good construct validity. The responsiveness of the QoL-ME, however, is poor.

Regarding the reliability of the QoL-ME, one of the additional modules (Leisure) has poor internal consistency (α = 0.5). Furthermore, four modules have questionable internal consistency (Support and attention, Lifestyle, Finances and Health and living; 0.7 > α ≥ 0.6). The internal consistency of the four remaining modules and of both core versions is acceptable to good (0.9 > α ≥ 0.7). Whilst these Alpha's are slightly lower than those of other QoL instruments are (5, 37), they hold up well when compared to similar multidimensional pictorial assessment scales. Engell et al. (41), for example, reported the results of a psychometric evaluation of a pictorial version of the Aachen Quality of Life Inventory (AQLI) targeting people with aphasia (41). The psychometric quality of the pictorial AQLI was evaluated by comparing results on the pictorial AQLI with scores on the regular AQLI that was filled out by the partners of a group of 24 patients. The results revealed notably lower reliability on some of the domains of the pictorial AQLI compared to the conventional AQLI that are very comparable to the QoL-ME (41). Second, Reid and colleagues (42) described the development and evaluation of a Pictorial Motivation Scale (PMS) targeting adolescents and adults with an intellectual disability. The PMS involves four subscales, whose Alpha's ranged between 0.64 and 0.88 (42). Moreover, the items of both the pictorial AQLI and the PMS involve both a picture and a verbal statement (41, 42), while the items of the additional modules of the QoL-ME consist solely of pictures.

The content of the QoL-ME was derived from a visual concept mapping study into the meaning of QoL for people with severe mental health problems (36). The use of concept mapping as the basis for measurement development is a dependable way to establish content validity (43). Two prior expectations regarding the validity of the QoL-ME were confirmed by the results. First, the domains of the core version for (forensic) psychiatric patients of the QoL-ME correlated highly (*r* = 0.55 −0.76) with the corresponding models of the MANSA. Second, scores on the language-based MANSA and the additional modules of the QoL-ME revealed a correlation of medium size (*r* = 0.3). Correlations between single items of the additional modules of the QoL-ME and corresponding MANSA items were of a similar magnitude, ranging between 0.15 and 0.39. These results are in accordance with the study of Engell and colleagues (41) in which they found correlations between the pictorial and language-based versions of the AQLI ranging between −0.01 and 0.75 but most correlations varied around 0.3 (41).

Further, the results reveal substandard responsiveness of the QoL-ME. An explanation for the inadequate external responsiveness of the QoL-ME may be provided by the literature on subjective well-being. In subjective well-being literature, a distinction between an affective and a cognitive component is firmly established (44–46). Research revealed that the two components of subjective well-being are determined by distinct variables and mechanisms (46). The pictorial approach to QoL assessment as employed in the additional modules of the QoL-ME may tap into the affective component of subjective well-being and QoL, whilst the language-based MANSA may draw on the cognitive component.

### Strengths and Limitations

In this study, the psychometric quality of the QoL-ME was assessed in a diverse sample including respondents from various cultural backgrounds and age groups, which is an important strength. Still, the results of this study should be regarded in light of four limitations. The first limitation concerns the convenience sampling method employed in this research. The resulting sample may not be representative for the target population, which may limit the generalizability of the results. Still, the aforementioned diversity in the sample indicate that the negative consequences of the employed sampling strategy are minimal. Second, analyses revealed that responders were significantly older than non-responders. Therefore, the findings related to the responsiveness of the QoL-ME may not be generalizable to younger age groups. However, we do not think this is a serious threat, because the results regarding the responsiveness of the QoL-ME are still inconclusive. The absence of clinical data collected in this study forms a third limitation. Scores on the QoL-ME cannot be related to the level of functioning or symptomatology of respondents, which is important when evaluating the usefulness of the QoL-ME in clinical practice. The fourth limitation relates to the absence of information on the occurrence of treatment interventions or life events known to influence the QoL of respondents during the study period. It is therefore unclear whether changes in the QoL of respondents are caused by treatment interventions, life events, inadequacies in the assessment instruments, or other causes.

### Future Research

The results of this study provide strong evidence for the suitability of the QoL-ME as an accessible alternative to existing language-based QoL instruments for people with severe mental health problems. At the same time, the multiple innovative characteristics of the QoL-ME, such as its flexible structure and visual approach to QoL assessment, offer a wide range of starting points for future research. First, future research may further investigate how the constituents of QoL may be optimally visualized, which may strengthen the psychometric quality of visual instruments such as the QoL-ME. Second, future research may investigate to what degree the visual assessment approach employed in the QoL-ME does indeed tap into the affective rather than cognitive component of QoL and what this means for the psychometric quality of the QoL-ME. Third, evaluating the psychometric characteristics of an instrument such as the QoL-ME that deviates from conventional instruments is challenging. Both the QoL-MEs' pictorial approach and its variable content make it difficult to find a suitable instrument for comparison. The results of this study provide an important first look into the psychometric quality of the QoL-ME. Additional research, however, is needed into the psychometric characteristics of the QoL-ME that involves alternative approaches, for example based on qualitative research methods or by using criterion variables that are known to be associated with quality of life. Fourth, future research that involves he clinical characteristics of respondents is needed to draw a more definitive conclusion regarding the suitability of the QoL-ME in clinical practice.

## Conclusion

This psychometric evaluation revealed adequate reliability and validity of the QoL-ME. Albeit slightly lower than the psychometric properties of conventional, language-based QoL instruments, in light of the psychometric quality of similar pictorial instruments, both the QoL-ME's reliability and validity can be considered sufficient. Overall, the QoL-ME displays adequate reliability and validity that is promising regarding the feasibility of its visual assessment approach. The responsiveness of the QoL-ME, however, is insufficient and additional research is required to evaluate and potentially modify the instrument to improve its responsiveness.

## Data Availability Statement

Research data will be stored at Tilburg University and comply with the quality infrastructure of Tilburg University. Data are managed and monitored with the required accuracy and organizational and technical measures to protect the processing of data has been taken. The research group, management board and Tranzo's (Tilburg University) quality professional will conduct quality checks on the data during the project to check if they are complete, correct and consistent. The research group will apply the recommended retention period for the data of at least 15 years. After completion of the project, Tilburg University will have governance over the final trial dataset, in compliance with their quality infrastructure. Any queries can be directed to the corresponding author.

## Ethics Statement

Ethical approval was obtained from the Ethics Committee of the Tilburg School of Behavioral and Social Sciences at Tilburg University (EC-2015.44). Written informed consent was obtained from each participant. All procedures performed in this study involving human participants were in accordance with the ethical standards of the institutional and/or national research committee and with the 1964 Helsinki Declaration and its later amendments or comparable ethical standards.

## Author Contributions

DB collected the data and wrote the first draft of the paper. CN supervised the execution of the research. DM, HO, and CN contributed in the process of drafting and revising. All authors were involved in the conception and design of the research and approve the content and all other aspects of the final version of this paper.

## Funding

This research was funded by the Netherlands Organization for Scientific Research, Grant/Award Number: 319-20-005. The funder had no role in, or ultimate authority over, the study design, data collection, management, data analysis, data interpretation, writing the report, and the final decision to submit the report for publication.

## Conflict of Interest

The authors declare that the research was conducted in the absence of any commercial or financial relationships that could be construed as a potential conflict of interest.

## Publisher's Note

All claims expressed in this article are solely those of the authors and do not necessarily represent those of their affiliated organizations, or those of the publisher, the editors and the reviewers. Any product that may be evaluated in this article, or claim that may be made by its manufacturer, is not guaranteed or endorsed by the publisher.

## Supplementary Material

Supplementary Material 1 Introductory video QoL-ME.mp4.

Supplementary Material 2 Video walkthrough QoL-ME.mp4.

Supplementary Material 3 Overview of visual content and item-scores.pdf.

## Abbreviations

AQLI: Aachen Quality of Life Inventory
LQoLP: Lancashire Quality of Life Profile
MANSA: Manchester Short Assessment of Quality of Life
MLQ: Meaning in Life Questionnaire
PMS: Pictorial Motivation Scale
QoL: Quality of Life
VA: Visual Analog Scale.
