# Supplementary material for: Psychometric Properties of the QoL-ME: A Visual and Personalized Quality of Life Assessment App for People With Severe Mental Health Problems
Source: Front Psychiatry. 2022 Jan 5;12:789704. doi: 10.3389/fpsyt.2021.789704 (PMC8767156; doi:10.3389/fpsyt.2021.789704)
Supplement: Supplementary Material 3 — Overview of visual content and item-scores.pdf. [file Data_Sheet_1.PDF]

Supplementary File 3. Overview of the content of the items of the additional modules of the QoL-ME. In addition, the mean scores at T0 are provided for every item and domain.

Introduction

Every item of the additional modules of the QoL-ME involves three images. For every item, respondents indicate their level of satisfaction using a 0-100 Visual Analogue Scale (VAS). The VAS scale involves visual anchors in the form of a satisfied and an unsatisfied emoticon. Respondents are requested to drag the green circle to the left or right, depending on their level of satisfaction with the depicted construct. Respondents can only advance to the next item after moving the green circle. Figure 1 below provides examples of two items. In the remainder of this additional file, every page provides the content of the items of one of the additional modules. Mean scores, including standard deviation and range, are also provided.

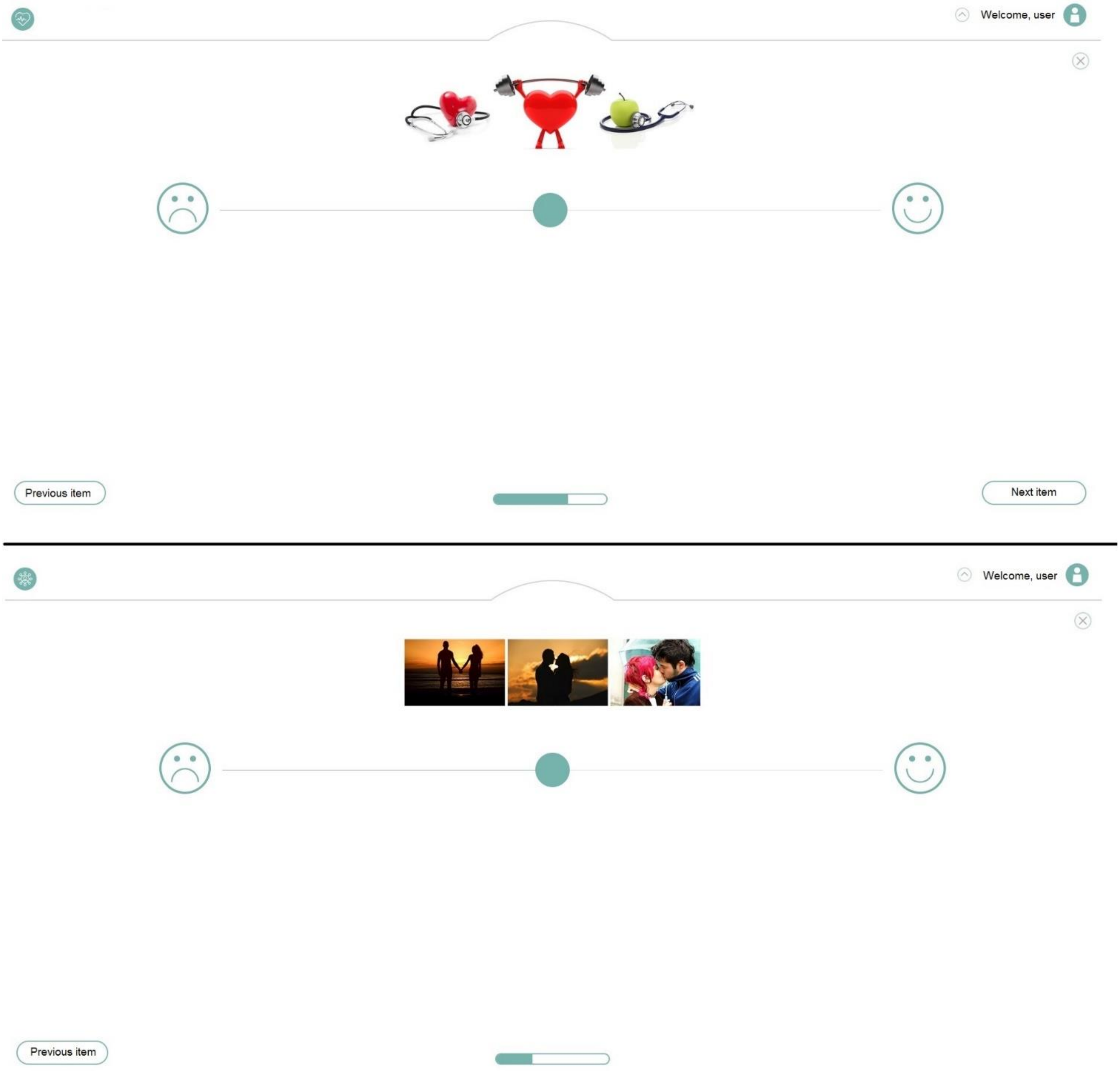

***Figure 1.** Example of two items of the additional modules of the QoL-ME.*

Supplementary File 3. Overview of visual content

Module 1: Support and attention (n = 101)

	Image 1	Image 2	Image 3	Mean VAS-score (SD)
**Item 1**	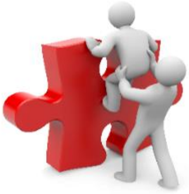	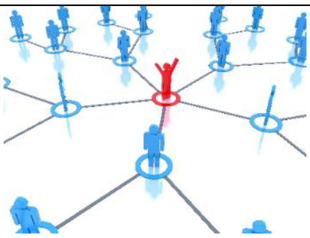	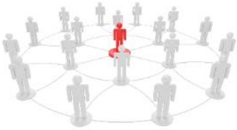	**74.34 (24.4), range = 0-100**
**Item 2**	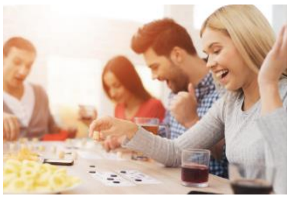	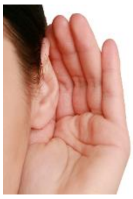 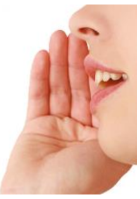	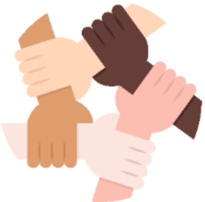	**73.86 (26.6), range = 3-100**
**Total**				**74.1 (21.9), range = 19.5-100**

Supplementary File 3. Overview of visual content

Module 2: Social relations (n = 97)

	Image 1	Image 2	Image 3	Mean VAS-score (SD)
**Item 1**	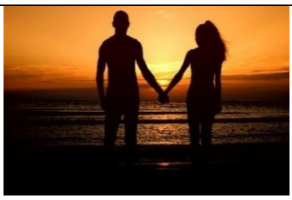	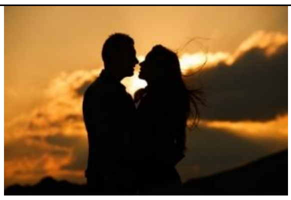	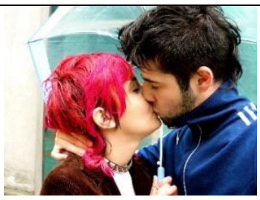	**70.5 (27.3),**<br>**range= 0-100**
**Item 2**	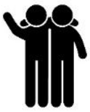	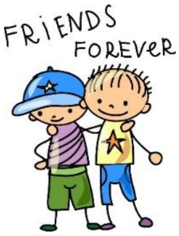	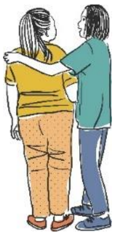	**75 (26.2),**<br>**range = 3-100**
**Item 3**	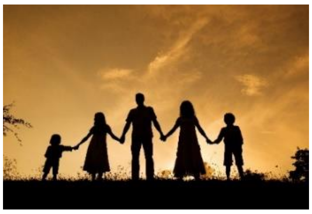	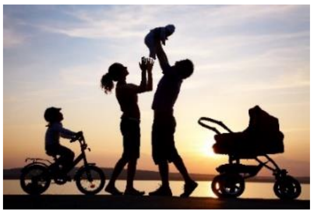	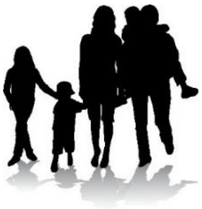	**72 (27.9),**<br>**range = 0-100**
**Item 4**	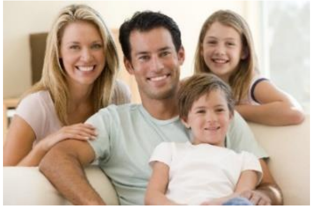	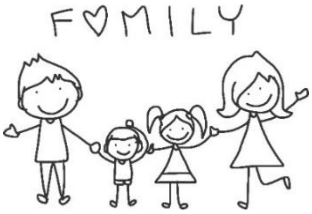	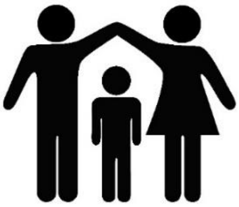	**75.2 (27),**<br>**range = 0-100**
**Total**				**73.16 (22.3),**<br>**range = 8-100**

Module 3: Happiness and love (n = 105)

	Image 1	Image 2	Image 3	Mean VAS-score (SD)
**Item 1**	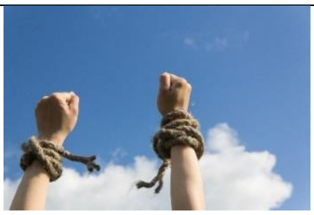	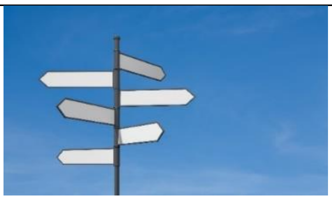	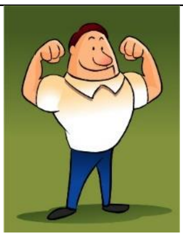	**68.46 (26.9),**<br>**range = 11-100**
**Item 2**	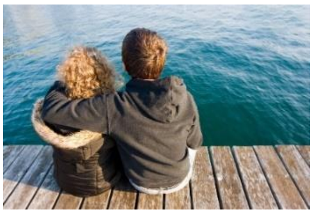	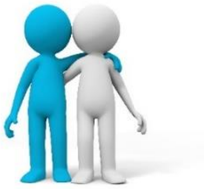	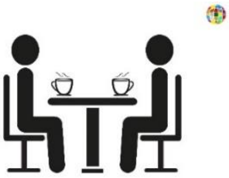	**75.06 (25),**<br>**range = 5-100**
**Item 3**	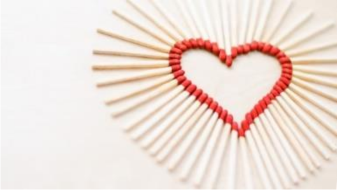	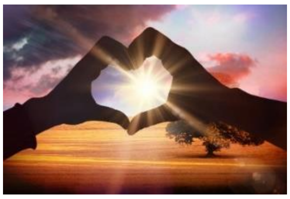	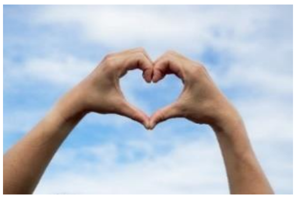	**76.1 (27.1),**<br>**range = 1-100**
**Item 4**	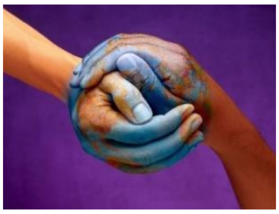	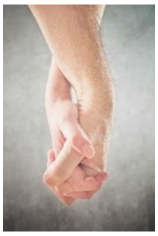	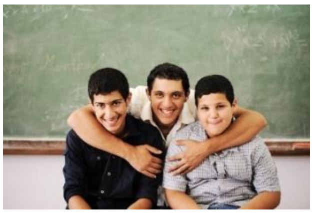	**76.8 (24.8),**<br>**range = 0-100**
**Total**				**74.1 (22.2),**<br>**range = 4-100**

Module 4: Relaxation and harmony (n = 111)

	Image 1	Image 2	Image 3	Mean VAS-score (SD)
Item 1	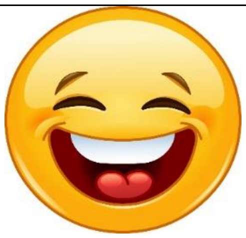	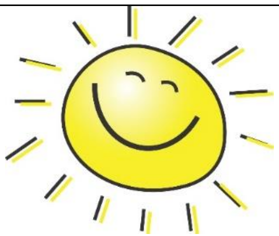	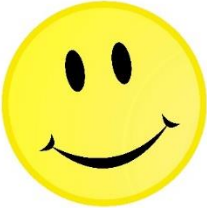	81.16 (21.1), range = 21-100
Item 2	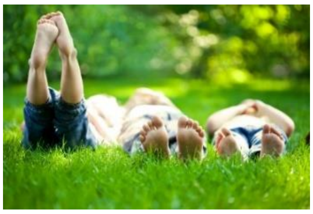	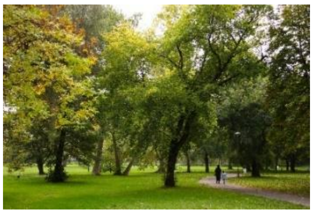	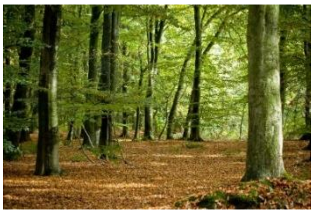	77.83 (23.6), range = 0-100
Item 3	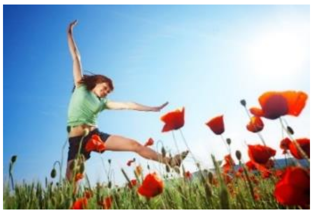	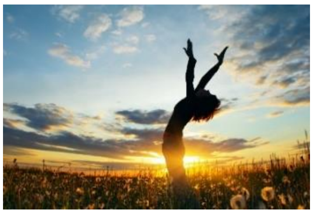	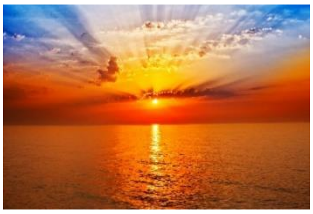	76.23 (25.9), range = 0-100
Item 4	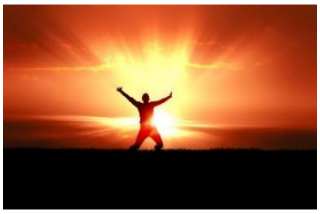	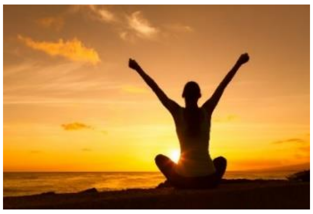	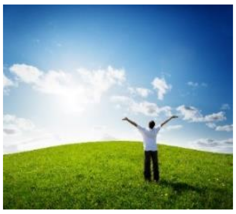	74.1 (25), range = 0-100
Total				77.34 (18.2), range = 25-100

Module 5: Leisure (n = 103)

	Image 1	Image 2	Image 3	Mean VAS-score (SD)
**Item 1**	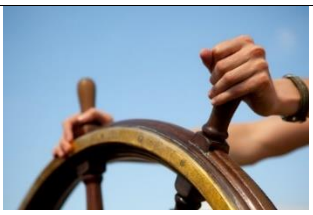	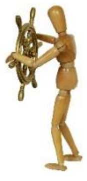	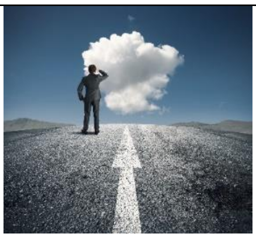	**70.6 (25.5),**<br>**range = 0-100**
**Item 2**	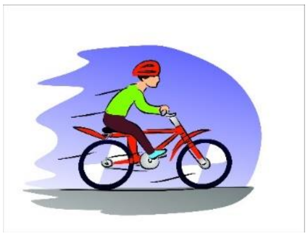	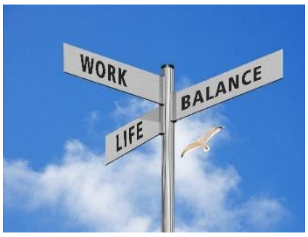	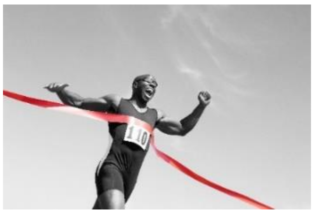	**73.37 (22.9),**<br>**range = 16-100**
**Total**				**72 (19.8),**<br>**range = 17.5-100**

Supplementary File 3. Overview of visual content

Module 6: Lifestyle (n = 97)

	Image 1	Image 2	Image 3	Mean VAS-score (SD)
**Item 1**	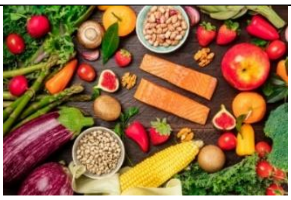	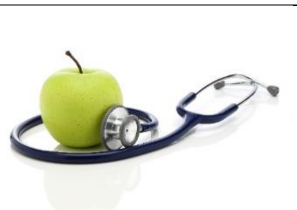	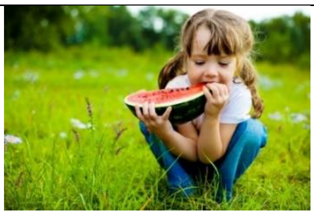	**76.8 (25.1),**<br>**range = 0-100**
**Item 2**	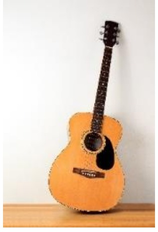	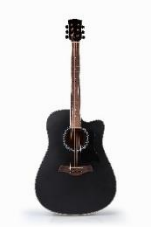	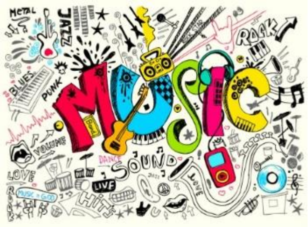	**78.6 (23.4),**<br>**range = 7-100**
**Item 3**	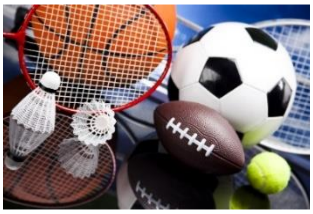	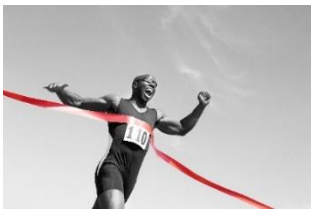	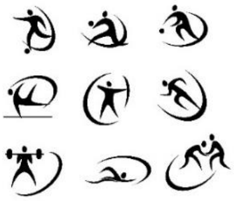	**72.6 (26.5),**<br>**range = 0-100**
**Total**				**76 (20.9),**<br>**range = 2-100**

Supplementary File 3. Overview of visual content

Module 7: Finances (n = 105)

	Image 1	Image 2	Image 3	Mean VAS-score (SD)
**Item 1**	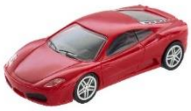	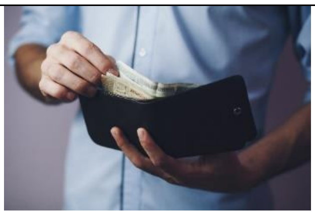	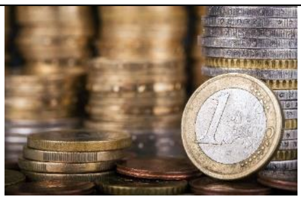	**64.64 (30.3), range = 0-100**
**Item 2**	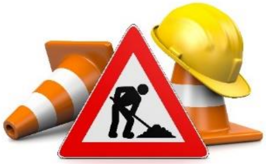	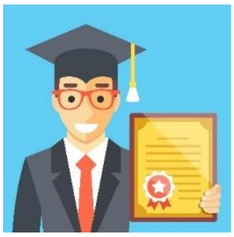	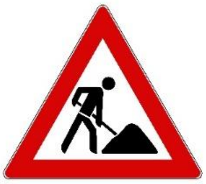	**60.8 (28.4), range = 1-100**
**Total**				**62.72 (26.7), range = 1-100**

Module 8: Health and living (n = 111)

	Image 1	Image 2	Image 3	Mean VAS-score (SD)
**Item 1**	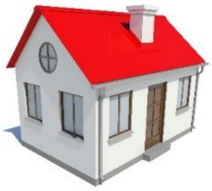	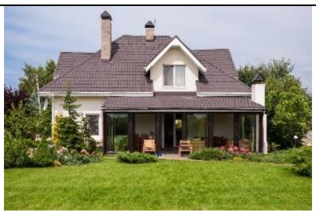	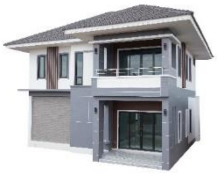	**69 (28),**<br>**range = 0-100**
**Item 2**	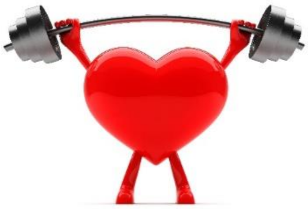	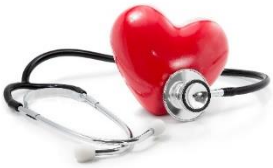	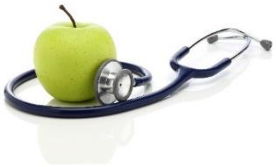	**71 (26.8),**<br>**range = 0-100**
**Total**				**70 (23.5),**<br>**range = 4-100**
